# Additive Manufacturing of Ti-48Al-2Cr-2Nb Alloy Using Gas Atomized and Mechanically Alloyed Plasma Spheroidized Powders

**DOI:** 10.3390/ma13183952

**Published:** 2020-09-07

**Authors:** Igor Polozov, Artem Kantyukov, Ivan Goncharov, Nikolay Razumov, Alexey Silin, Vera Popovich, Jia-Ning Zhu, Anatoly Popovich

**Affiliations:** 1 Peter the Great St. Petersburg Polytechnic University, Polytechnicheskaya 29, 195251 St. Petersburg, Russia; kantyukov.artem@mail.ru (A.K.); goncharov_is@spbstu.ru (I.G.); n.razumov@onti.spbstu.ru (N.R.); silin_ao@spbstu.ru (A.S.); director@immet.spbstu.ru (A.P.); 2 Department of Materials Science and Engineering, Delft University of Technology, 2628 Delft, The Netherlands; V.Popovich@tudelft.nl (V.P.); J.Zhu-2@tudelft.nl (J.-N.Z.)

**Keywords:** selective laser melting, additive manufacturing, titanium alloy, microstructure, mechanical alloying

## Abstract

In this paper, laser powder-bed fusion (L-PBF) additive manufacturing (AM) with a high-temperature inductive platform preheating was used to fabricate intermetallic TiAl-alloy samples. The gas atomized (GA) and mechanically alloyed plasma spheroidized (MAPS) powders of the Ti-48Al-2Cr-2Nb (at. %) alloy were used as the feedstock material. The effects of L-PBF process parameters—platform preheating temperature—on the relative density, microstructure, phase composition, and mechanical properties of printed material were evaluated. Crack-free intermetallic samples with a high relative density of 99.9% were fabricated using 900 °C preheating temperature. Scanning electron microscopy and X-Ray diffraction analyses revealed a very fine microstructure consisting of lamellar α_2_/γ colonies, equiaxed γ grains, and retained β phase. Compressive tests showed superior properties of AM material as compared to the conventional TiAl-alloy. However, increased oxygen content was detected in MAPS powder compared to GA powder (~1.1 wt. % and ~0.1 wt. %, respectively), which resulted in lower compressive strength and strain, but higher microhardness compared to the samples produced from GA powder.

## 1. Introduction

Gamma TiAl-based intermetallic alloys are attractive materials for structural high-temperature applications due to their high specific strength at room and elevated temperatures, good creep, and oxidation resistance [1]. These properties make them promising candidates for replacing nickel-based superalloys in gas turbine engines [2]. One of the most widely known γ-TiAl alloys is the Ti-48Al-2Cr-2Nb (at. %) alloy, which is currently used by General Electric for low-pressure turbine blades [3,4]. While TiAl alloys possess high strength, their poor ductility and brittleness at room temperatures severely complicate their processability by conventional manufacturing techniques and limit their application [5]. Casting followed by hot isostatic pressing (HIP) has been used to conventionally fabricate TiAl alloy parts [6]; however, this approach has its limitations in terms of high cost and design flexibility.

In recent years, additive manufacturing (AM) has been applied to manufacture various titanium alloys [7,8,9] as well as titanium aluminide alloys [10,11,12]. AM is a promising way to manufacture intermetallic alloy parts since it offers significant advantages in terms of design freedom and cost reduction compared to conventional methods. However, high cooling rates typical for laser-based powder bed fusion and directed energy deposition AM techniques lead to high residual stresses, which makes it difficult to produce crack-free intermetallic parts. Shi X. et al. [13] showed that it is not possible to produce crack-free Ti-47Al-2Cr-2Nb alloy samples by the laser powder bed fusion (L-PBF) using 200 °C preheating. The L-PBF of Ti-48Al-2Cr-2Nb alloy with 450 °C preheating resulted in severe cracking formation, indicating that higher preheating temperatures must be utilized [14]. Selective electron beam melting (SEBM) has been proved feasible in fabrication of TiAl alloys [15,16,17]. Utilizing an electron beam to preheat the powder bed to temperatures around 1000 °C allows one to drastically reduce residual stresses and suppress crack formation during the fabrication of TiAl alloys. One disadvantage of SEBM process is its much lower geometrical precision compared to L-PBF process. High-temperature powder bed preheating is required to produce crack-free TiAl alloys using the L-PBF process. Platform preheating temperatures of 800–1000 °C with inductive heating have been successfully used to obtain crack-free samples during the L-PBF of titanium aluminides [18,19]. However, microstructure and mechanical properties are still underexplored and require extensive characterized to evaluate the feasibility of L-PBF for manufacturing of TiAl alloys.

Another concern for further development of AM TiAl is the commercial availability of intermetallic alloys powders with properties suitable for their application in AM. Gas and plasma atomization are currently the most common processes used for the fabrication of spherical powders for L-PBF or SEBM technologies [20,21]. While atomization techniques allow manufacturing of spherical powders, these technologies are rather costly, especially considering the complex compositions of intermetallic alloys. Mechanical alloying (MA), followed by the plasma spheroidization (PS) process, is an alternative approach to obtain spherical powders with reduced costs for application in AM [22,23]. Irregular MA powders are treated in a high-temperature plasma jet, resulting in rapid melting and solidification and leading to spherical powders [24]. Application of MA plasma spheroidized (MAPS) powders in L-PBF or SEBM processes can lead to lower cost of these techniques. However, the microstructure and properties of alloys fabricated by AM from MAPS powders are yet to be evaluated and compared to gas atomized (GA) powders to assess the feasibility of such an approach.

The objective of this paper is to evaluate the feasibility of L-PBF process to fabricate crack-free Ti-48Al-2Cr-2Nb alloy from different feedstock powders and using an inductive high-temperature platform preheating. Two types of powders are used: a commercially available GA powder and an in-house produced MAPS TiAl-alloy powder. The effects of L-PBF process parameters and preheating temperature on fabricated TiAl-alloy microstructure and mechanical properties are investigated.

## 2. Materials and Methods

### 2.1. Materials

Two types of powders were used in the study to fabricate the samples by L-PBF process. The GA powder of Ti-48Al-2Cr-2Nb alloy supplied by AMC Powders (Beijing, China) and produced by electrode induction gas atomization (EIGA) had the following particle size distribution: d_10_ = 17.4 µm, d_50_ = 33.8 µm, d_90_ = 60.5 µm. Further description of the powder’s composition and morphology is presented in Section 3.1. The second powder of TiAl-alloy was produced in-house by MA and PS processes. Preliminary experiments of the MA process were carried out using a Fritsch Pulverisette 4 planetary mill by milling the elemental powders of Ti, Al, Cr, and Nb (with 99.9% purity) blended with the proportion of Ti-48Al-2Cr-2Nb (at. %) alloy. A more detailed description of the MA process and characterization of the powders can be found in [25]. A dry grinding SD5 laboratory attritor produced by Union Process (Akron, OH, USA) was used for MA. The elemental powder blend was milled for 12 h in an argon atmosphere with the rotation speed of 270 rpm and 20:1 ball to powder mass ratio using stainless steel balls with 10 mm diameter. After the MA process, the powder was sieved to 0–71 µm fraction and subjected to PS using the TEK-15 system (Tekna, Sherbrooke, QC, Canada). The Ar-He gas was used as the plasma forming gas. The powder feeding rate was set to 15 g/min and the plasma torch power was 15 kW. The final particle size distribution of the MAPS powder was the following: d_10_ = 9.7 µm, d_50_ = 33.3 µm, d_90_ = 67.7 µm.

### 2.2. Laser Powder-Bed Fusion

The L-PBF process was carried out using AconityMIDI (Aconity3D GmbH, Herzogenrath, Germany) system. The system is equipped with a 1070 nm wavelength fiber laser with a maximum power of 1000 W. Cylindrical samples with a 10 mm diameter and 10 mm height were fabricated for further investigation. The samples were fabricated on a Ti-6Al-4V substrate, which was put on a molybdenum platform. The molybdenum substrate was inductively preheated to a set temperature, which was continuously controlled by a thermocouple under the molybdenum platform. The titanium substrate was then conductively heated by the molybdenum substrate before starting the L-PBF process. The process chamber was continuously flooded with high purity argon gas to achieve oxygen content in the chamber below 20 ppm. After the build process was finished, the platform and the samples were cooled to room temperature with a cooling rate of approximately 5 °C/min.

The platform preheating temperature was varied from 600 to 900 °C and the scanning speed (S) was varied from 650 to 1250 mm/s, while the laser power (P), hatching distance (HD), and layer thickness (L) were set to a fixed value for most of the samples. The laser spot diameter was set to ~80 µm for most of the samples. A chessboard scanning pattern with 5 × 5 mm^2^ squares and a rotation angle of 67 °C was used for the L-PBF process. The L-PBF processing parameters used to produce the samples from the MAPS and the GA powders are shown in Table 1. The values were chosen based on the preliminary results and the published data on L-PBF of TNBV4 alloy [26].

One sample was fabricated using GA powder and a large laser spot diameter of 500 µm (further denoted as “Large spot”) and increased laser power and layer thickness values, while the volume energy density (VED) was at a similar value as for the samples produced with 80 µm spot diameter (see Table 1). The larger spot was used in order to investigate how the microstructure of a TiAl-alloy can be varied by using an increased laser spot diameter coupled with high laser power and increased layer thickness. As demonstrated in the previous study, L-PBF processing with increased power energy can result in a strongly textured coarse microstructure, as shown in the case of Inconel 718 alloy [27,28].

### 2.3. Characterization

The as-fabricated samples were cut and polished along the build direction (BD) for the microstructural characterization. Mira 3 LMU (TESCAN, Brno, Czech Republic) scanning electron microscope (SEM) in the backscattered electrons (BSE) mode was utilized to evaluate the microstructures of non-etched samples. Energy Dispersive Spectroscopy (EDS) was used for the chemical analysis of the samples and powders on the polished cross-sections.

The phase composition of the powders and the fabricated samples was analyzed with a Bruker D8 Advance X-ray diffraction (XRD) (Bruker, Germany) using Cu-Kα (λ = 1.5418 Å) irradiation.

The relative density was measured by a standard metallographic technique, which includes taking a minimum of five different locations of the polished samples with an optical microscope (OM) Leica DMI5000 () at 50× magnification. The OM images were then used to isolate the pores from the bulk material (Leica, Wetzlar, Germany) using ImageJ software. The calculated fraction of the image defined as bulk materials was used as the relative density value. General Electric (Boston, MA, USA) Phoenix Vtomex Computed Tomography (CT) System was used for X-ray microtomography analyses (CT) of the samples with a voxel size of 10 μm. Avizo software was used to visualize the CT-data and evaluate the porosity of the samples.

The oxygen content in the powder and the fabricated samples was measured using the inert-gas fusion-infrared (IGF) method with a LECO TC-500 analyzer (LECO, St Joseph, MI, USA).

The microhardness of the samples was measured using a Buehler VH1150 testing machine with 300 g load and 10 s dwell time.

L-PBF process parameter sets that produced the sample with the highest relative density were then used to fabricate cylindrical samples with 4 mm diameter and 20 mm height for the compression tests. The samples were cut by electrical discharge machining to achieve 5 mm height. Room temperature compression tests were performed using a universal testing machine (Zwick/Roell Z100, Ulm, Germany) with a strain rate of 0.1 mm/min. A minimum of three samples per point were tested.

## 3. Results and Discussion

### 3.1. Powder Characterization

**Figure 1:**
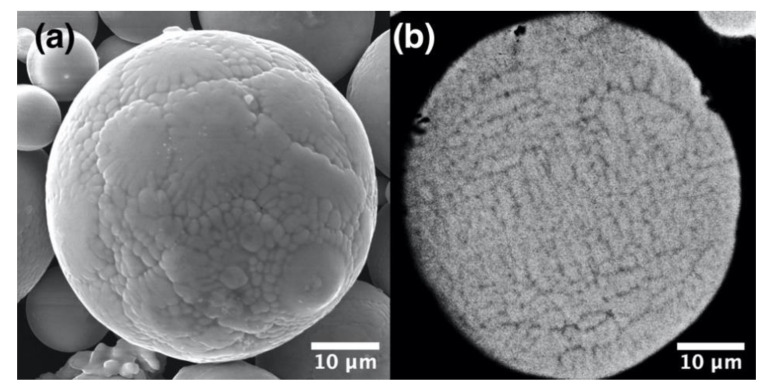
Scanning electron microscope (SEM) images of the gas atomized powder (GA) showing (**a**) surface morphology and (**b**) cross-section of a particle.

The particle size distribution of gas atomized (GA) powder was found to be d_10_ = 17.4 µm, d_50_ = 33.8 µm, d_90_ = 60.5 µm. As can be seen in Figure 1, the GA particles have a spherical shape and dendritic surface morphology, while the cross-section shows a typical for the GA process dendritic microstructure [29,30]. The chemical composition of the powder is shown in Table 2.

**Figure 2:**
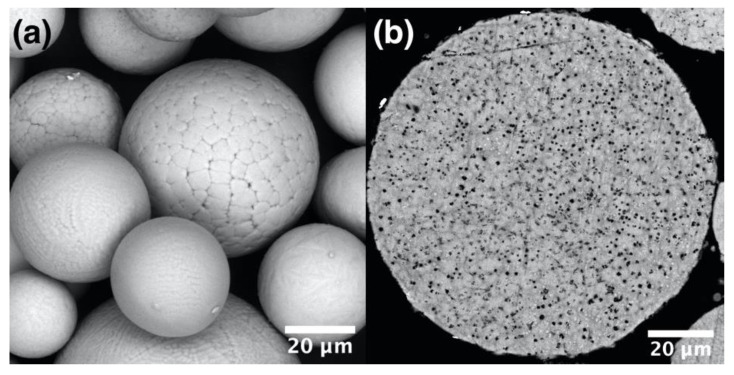
SEM images of the mechanically alloyed plasma spheroidized (MAPS) powder showing (**a**) surface morphology and (**b**) cross-section of a particle.

The particle size distribution of mechanical alloyed plasma spheroidized (MAPS) powder was found to be d_10_ = 9.7 µm, d_50_ = 33.3 µm, d_90_ = 67.7 µm. The particles also have a spherical shape and dendritic surface morphology (Figure 2a). A significant difference from the GA powder is the presence of small oxides inside the particles, which can be seen as black precipitates in the cross-section image (Figure 2b). The presence of oxides can be attributed to an increased oxygen content as shown in Table 2. The oxygen pickup most likely occurred during the MA process. Al was partially lost during the PS process. Thus, further optimization of the process may be carried out to obtain a proper chemical composition.

**Figure 3:**
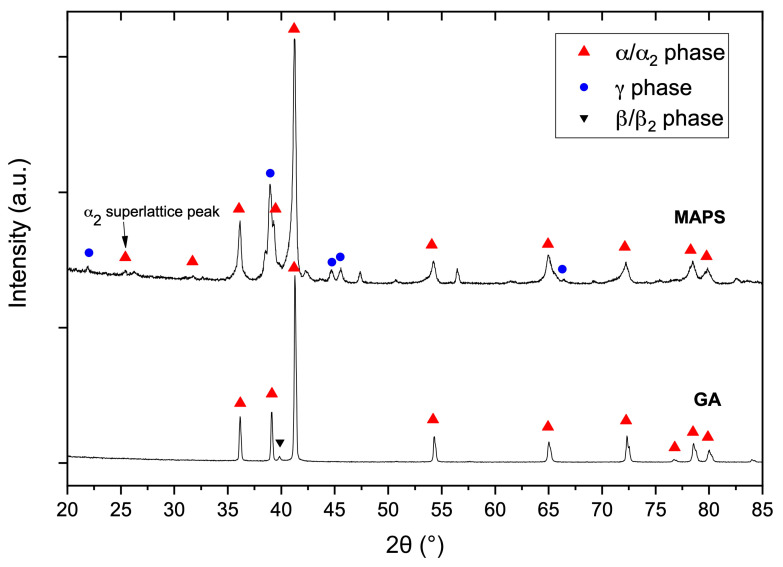
X-ray diffraction (XRD) patterns of MAPS and GA powders.

The XRD pattern of the GA powder shows peaks corresponding to α/α_2_-phase with a hexagonal lattice and a small peak corresponding to β-phase with a body-centered cubic (BCC) lattice (Figure 3). The GA process is characterized by high cooling rates during the solidification of powders preserving the α/α_2_-phase. The phase composition of MAPS powder is characterized by the presence of α_2_ (Ti3Al-based) and γ (TiAl-based) phases. The presence of the ordered α_2_-phase instead of α-phase can be confirmed by identifying the most intense superlattice α_2_ peak [31], which is seen in the case of the MAPS powder. The difference in the phase composition between GA and MAPS powders might be the result of Al and O content differences. As shown in [32], an increase of oxygen content can decrease the volume fraction of γ-phase and increase the volume fraction of hexagonal α/α_2_-phase.

### 3.2. Densification via Laser Powder-Bed Fusion

**Figure 4:**
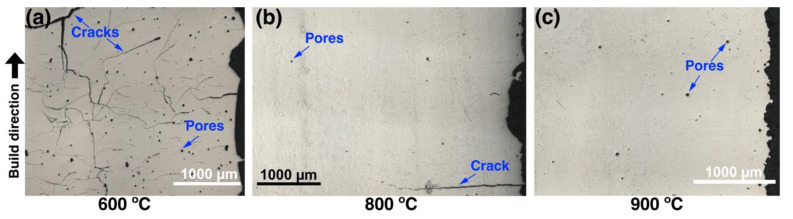
Optical images of the cross-sections of the samples fabricated from MAPS powder. The images show representative cross-sections of the samples obtained using different preheating temperatures: (**a**) 600 °C (sample 1_600), (**b**) 800 °C (sample 1–800), (**c**) 900 °C (sample 1_900).

Figure 4 shows the effect of platform preheating temperature on the formation of cracks in samples fabricated from the MAPS powder. As can be seen in Figure 4a, severe cracking is observed when a relatively low preheating temperature of 600 °C was used during the L-PBF process. Increasing the preheating temperature to 800 °C significantly reduced the number of cracks; however, occasional horizontal cracks perpendicular to the BD were still present. Further increase in the preheating temperature up to 900 °C resulted in elimination of cracks. This is in agreement with the brittle-ductile transition temperature (BDTT) of the TiAl-alloy, which is around 750–780 °C [33]. Increased ductility above the BDDT allows the material to accommodate high stresses during L-PBF and avoid cracking. Similar results were obtained in [18] when the Ti-44.8Al–6Nb–1.0Mo–0.1B (at. %) powder was processed by L-PBF at 800 °C platform preheating. It should be noted that occasional horizontal cracks were still present on the edges of the specimen, likely due to a high thermal gradient (Figure 4b). Similar results in terms of cracks formation were obtained for the GA powder. Further investigation of the relative density was carried out only on the samples fabricated at 800 and 900 °C preheating temperatures.

**Figure 5:**
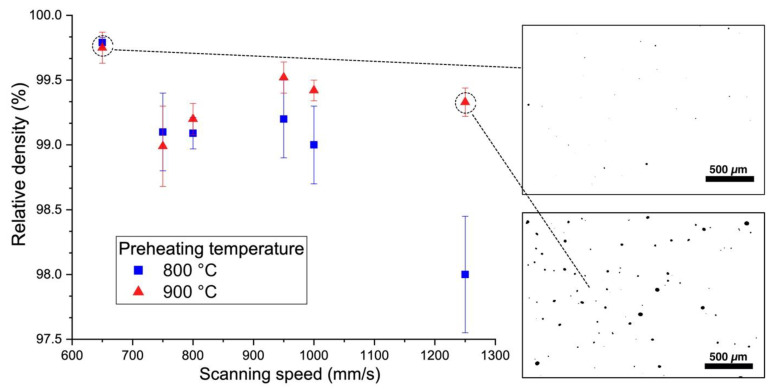
Effect of scanning speed and platform preheating temperature on the relative density of the samples fabricated from MAPS powder with cross-section images showing typical pore distribution.

**Figure 6:**
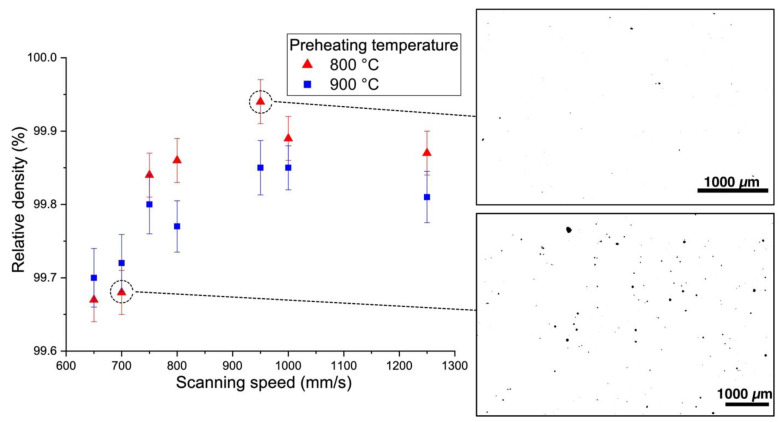
Effect of scanning speed and platform preheating temperature on the relative density of the samples fabricated from GA powder with cross-section images showing typical pore distribution.

Figure 5 and Figure 6 show the effect of scanning speed on the relative density of the samples fabricated using the MAPS and the GA powders, respectively. In the case of the MAPS powder, the highest relative density (99.75 ± 0.05%) was achieved at 650 mm/s scanning speed (which corresponds to 70 J/mm^3^ VED) for both 800 and 900 °C preheating temperatures. Using the GA powder resulted in an overall higher relative density values (all values were higher than 99.6%). The highest relative density of 99.94 ± 0.05% was obtained at 800 °C preheating temperature and 950 mm/s scanning speed which corresponds to 48 J/mm^3^ VED. In general, a higher preheating temperature resulted in a slightly higher porosity, which suggests that some overheating might have taken place during the L-PBF and led to formation of keyhole pores. At the same time, applying high scanning speed of 1250 mm/s resulted in an increased porosity for both powders, which might be the result of insufficient melting or melt pool instability [34,35].

**Figure 7:**
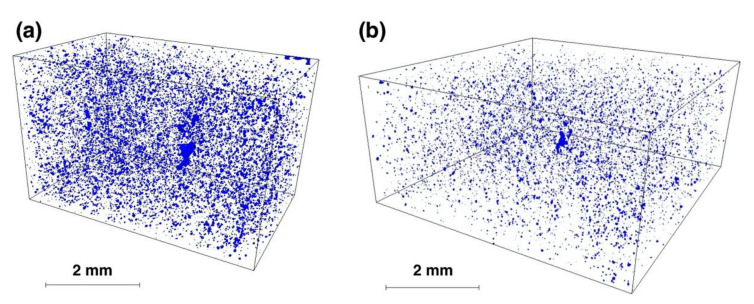
Computer tomographic reconstruction of the porosity volume in the samples produced from (**a**) MAPS powder (sample 2_800) and (**b**) GA powder (sample 5_800).

The samples fabricated from the MAPS and the GA powders having the highest relative densities were taken further for the CT-investigation. The results (Figure 7) showed that the median size of the pores was around 40 µm for the MAPS sample and around 15 µm for the GA sample. The porosity volume was visibly lower in the case of the GA sample compared to the MAPS sample, which is compliant with the metallographic measurements. The pores were found to be mostly spherical, suggesting that these are gas pores formed due to entrapped gas originating from the argon gas or melt pool vaporization [36]. A few lack-of-fusion defects were also found for both samples, indicating poor bonding defects or incomplete melting of some powders [37]. According to the CT-results, the GA sample had porosity volume less than 0.1%, while the MAPS sample had porosity volume of around 0.3%. Higher porosity in MAPS samples might be attributed to an increased oxygen in the feedstock powder, as well as the initial porosity from the powder. This correlates with the results obtained by Li et al. [38], where they showed that a powder with lower oxygen content resulted in better densification during L-PBF of 316L alloy.

### 3.3. Microstructural Characterization

**Figure 8:**
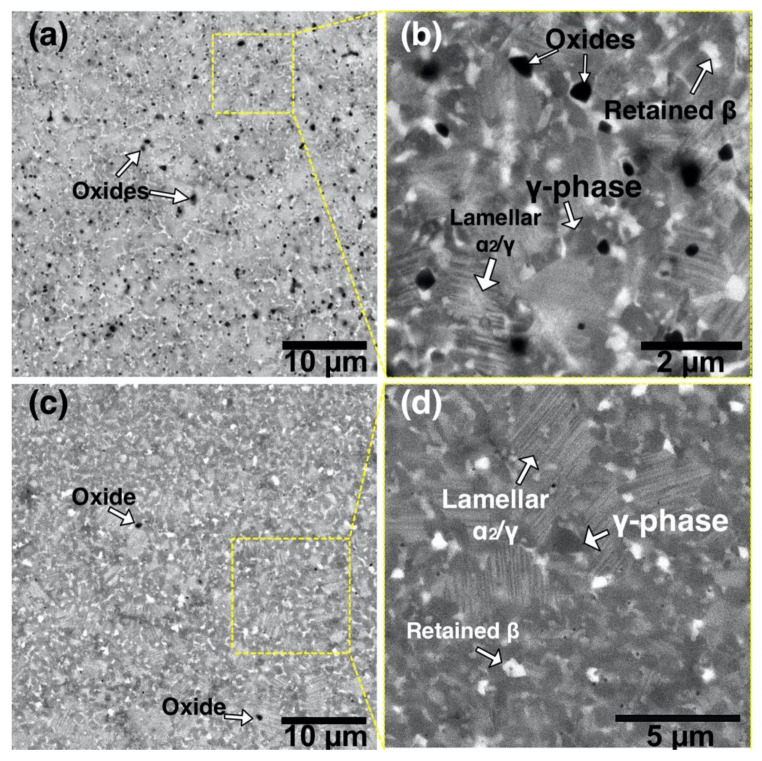
BSE-SEM images showing microstructures of the samples fabricated from the MAPS powder at (**a**,**b**) 800 °C (sample 2_800) and (**c**,**d**) 900 °C preheating temperature (sample 2_900).

**Figure 9:**
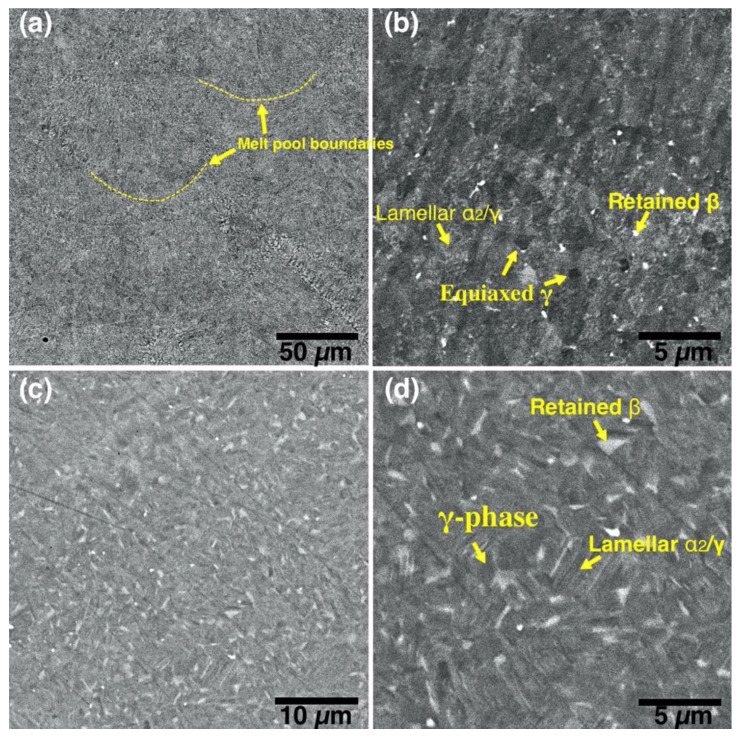
Backscattered electrons (BSE) SEM images showing microstructures of the samples fabricated from GA powder at (**a**,**b**) 800 °C (sample 5_800) and (**c**,**d**) 900 °C preheating temperature (sample 5_900).

Figure 8 shows microstructure of the TiAl-alloy fabricated from MAPS powder at 800 °C (Figure 8a) and 900 °C (Figure 8b) preheating temperatures. According to the Ti-Al phase diagram [39], these temperatures correspond to the α_2_+γ phase field. In both cases, the alloy has a very fine duplex microstructure consisting of lamellar α_2_/γ colonies (gray), equiaxed γ grains (dark gray), and residual β phase (white) as confirmed by the XRD results (Figure 9). Retained β-phase was also found in the microstructure of the SEBM-processed Ti-48Al-2Cr-2Nb alloy in the paper [10]. While the Ti-48Al-2Cr-2Nb is an α-solidifying alloy, high cooling rates during the L-PBF process can induce solidification of a metastable β-phase. As shown in Table 3, there is Al loss of about 2–3 at. % in the samples fabricated from MAPS powder compared to the initial powder. Reducing the Al concentration to about 40–41 at. % leads to the solidification through the Ti-rich side of the peritectic reaction and formation of the β-phase. The retained β-phase in the TiAl-alloy can increase its ductility, however the strength at room and elevated temperatures can be worsened due to refractory elements segregation and reduced solid solution strengthening in the lamellar regions [40]. Thus, a subsequent heat treatment should be considered to transform the metastable β-phase in the TiAl alloy.

**Figure 10:**
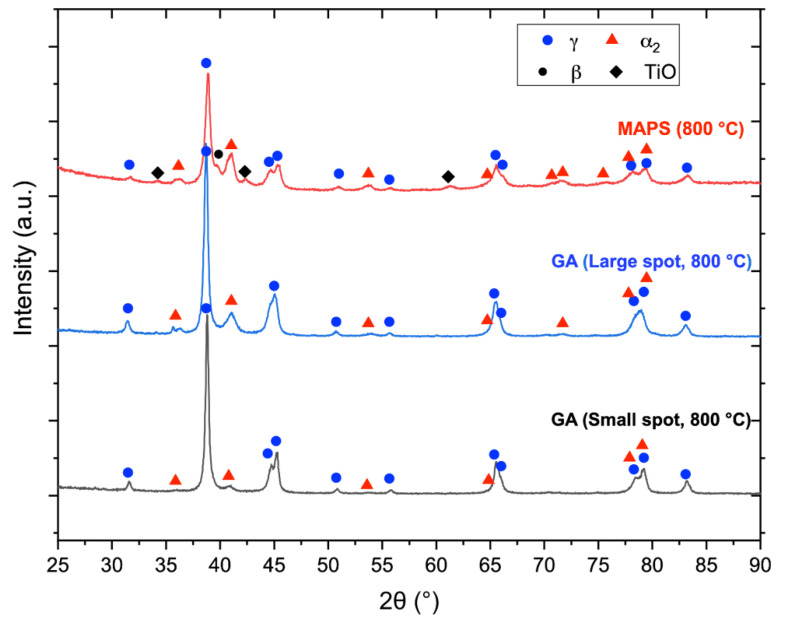
XRD patterns of the samples fabricated from GA and MAPS powders.

According to the XRD results (Figure 10), the alloy has some oxides corresponding to the TiO phase. The oxides mostly likely originated from the initial powder; however, some oxygen pickup during the L-PBF process could take place as well. The oxygen measured by LECO analysis showed that samples fabricated from MAPS powder had oxygen content around 1.3 wt. %, while samples fabricated from GA powder had 10 times lower oxygen content (~0.14 wt. %).

Figure 9 shows microstructures of the samples fabricated from GA powder at 800 °C and 900 °C preheating temperatures. The obtained microstructure is similar to MAPS samples and mainly consists of lamellar α_2_/γ colonies, equiaxed γ grains, and the retained β phase. Crescent-shaped melt pool boundaries with a width of about 80–90 µm can be found in the microstructure as shown in Figure 9a. There were no oxide precipitates found in the case of GA powder due to relatively low oxygen content in the initial powder. Compared to the MAPS samples, the amount of retained β phase is visibly lower in GA samples since their Al content is higher (as can be seen from Table 3 and Table 4) and the solidification is expected to take place mainly through the α phase region. However, when the preheating temperature was increased to 900 °C from 800 °C the amount of β phase visibly increased.

The microstructure of the samples fabricated from GA powder shows fewer lamellar α_2_/γ colonies compared to the MAPS samples. This could due to lower oxygen content in the GA samples since a decreased oxygen content leads to an increased γ-phase volume fraction in TiAl-alloys [32]. XRD results (Figure 10) confirmed that samples fabricated from GA powder consist mainly of γ phase (TiAl) and α_2_ (Ti_3_Al) phase.

**Figure 11:**
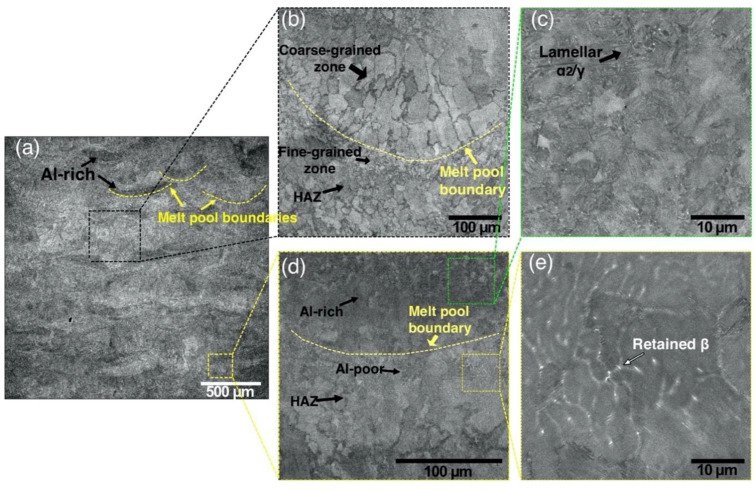
BSE-SEM images showing the microstructure variation in the “Large spot” sample produced from GA powder: (**a**) a general view, (**b**) a magnified area showing a melt pool section, (**c**) Al-rich area, (**d**) a melt pool boundary, (**e**) Al-poor area

Figure 11 shows the microstructure of the “Large spot” sample fabricated from the GA powder using a laser spot diameter of about 500 µm. In this case, the melt pool width was roughly about 500–600 µm as can be seen in Figure 11a. Larger melt pool during the L-PBF process is expected to result in lower cooling rates and, subsequently, a coarser microstructure [41]. The obtained microstructure is highly inhomogeneous and consists of various layered regions. There are coarse columnar γ grains inside the melt pool growing towards the middle of the melt pool, as can be seen in Figure 11b. A fine-grained zone can be seen underneath the melt pool boundary, which likely formed during recrystallization in a heat-affected zone (HAZ). The L-PBF involves repetitive heating and cooling resulting in a complex thermal history. This leads to intrinsic non-uniform heat treatment during the L-PBF process. A similar alternative-band microstructure of the Ti-47Al-2Cr-2Nb alloy was observed after direct laser deposition in [42] and the authors attributed it to the effect of cyclic heat treatment derived from multiple laser exposure. The deposition and laser exposure of the next layers leads to heating the underlying solidified material. In this HAZ the material reaches α-transus temperature, then cools down into α+γ region resulting in a refined microstructure [10].

Al-rich and Al-poor regions can be distinguished from the BSE-SEM images of the “Large spot” sample. Al-rich zone is seen at the bottom of a melt pool as a dark band while the Al depletion is observed at the top of the melt pool, as can be seen in Figure 11d. It suggests that the Al loss occurred mostly closer to the surface of a melt pool. The overall Al loss in “Large spot” sample was around 3 at. % compared to the initial powder (Table 4). Al segregation in the L-PBF-processed Ti-6Al-4V alloy samples was observed in [43] resulting in dark bands at the bottom of a melt pool. Similarly, SEBM resulted in a banded, inhomogeneous microstructure of a γ-TiAl based alloy due to the vaporization of Al [44]. Al-rich regions consist of lamellar α_2_/γ colonies as can be seen in Figure 11c, while Al-poor regions have lamellar colonies surrounded by the retained β phase as shown in Figure 11e. The differences in microstructures between Al-rich and Al-poor areas are in a good agreement with the Ti-Al phase diagram [45].

### 3.4. Mechanical Properties

Table 5 presents the results of room temperature compressive tests of the samples fabricated using GA and MAPS powders. High ultimate compressive strength values were obtained for both GA and MAPS samples, which were 2277 ± 71 MPa and 1910 ± 37 MPa, respectively. The compressive strain values were around 32%–35% for the GA samples and 15%–17% for the MAPS. Using the GA powder resulted in both higher compressive strength and strain. This can be explained by an increased oxygen content in MAPS powder, which is known to embrittle TiAl-alloys [46], as well as a higher β-phase content in MAPS samples, which is softer than intermetallic γ and α_2_ phases [47] and can reduce the strength of the alloy. The overall room temperature compressive performance of the samples fabricated by L-PBF with a high-temperature preheating showed promising results and exceeded the compressive strength of SEBM samples and showed significantly better results than the samples fabricated by L-PBF with a non-preheated platform [48]. The GA samples also showed superior compressive performance compared to the conventional TiAl-alloy [49] and SEBM Ti-48Al-2Cr-2Nb alloy [50].

**Figure 12:**
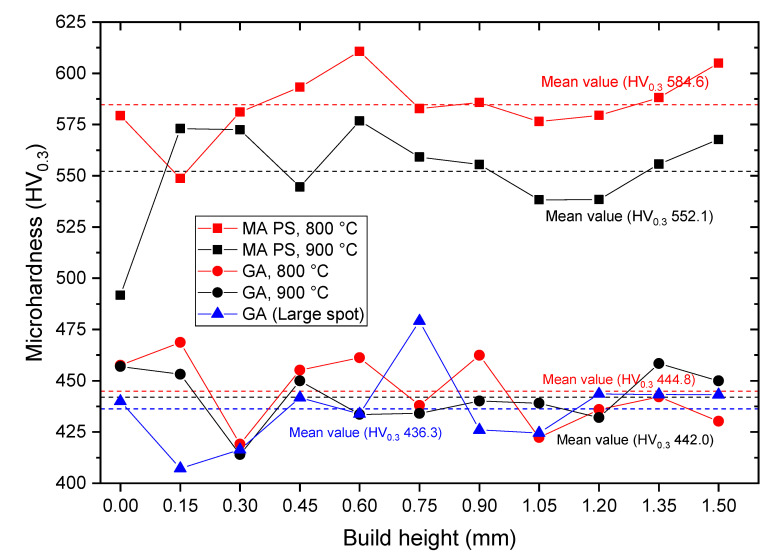
Microhardness variation along the build direction of the samples manufactured from MAPS powder and GA powder. The dashed lines correspond to the mean values: red—for the samples fabricated at 800 °C, black—at 900 °C, blue—using the large laser spot size.

The microhardness variation over a range of 1.5 mm along the BD was measured for different samples, as shown in Figure 12. All samples demonstrated a fluctuating microhardness profile, which is associated with the microstructural inhomogeneity. A similar microhardness variation along the BD was obtained in [42] for the direct laser deposited Ti-47Al-2Cr-2Nb alloy, with an average value of 400 HV. The highest mean microhardness values were obtained for the samples fabricated from MAPS powder: 584 HV and 552 HV for 800 °C and 900 °C preheating temperatures, respectively. This correlates well with the oxygen content in the samples: higher microhardness was obtained for the samples with higher oxygen content. As shown in [51] for the Ti-48Al-2Cr-2Nb alloy, an increase in oxygen content increases α_2_ phase volume fraction that has higher hardness compared to γ phase. At the same time, decreased Al content in MAPS samples could prevent the formation of γ-phase, resulting in a higher α_2_-phase volume fraction and higher microhardness.

The samples fabricated from GA powder showed mean microhardness around 440 HV, which is higher than as-fabricated SEBM (253 HV) and conventional (371 HV) TiAl-alloy [50]. Higher microhardness might be attributed to a very fine microstructure in the case of the L-PBF. High microhardness can be beneficial for wear performance [52]; however, it also suggests increased brittleness and reduced ductility [51]. Thus, a more complex mechanical properties characterization is recommended.

## 4. Conclusions

In this work it was demonstrated that pre-alloyed spherical GA and MAPS powders of TiAl-based alloy can be used to fabricate crack-free samples using the L-PBF process with high-temperature platform preheating. The following main conclusions were drawn:

1. Crack-free samples were fabricated with 900 °C platform preheating temperature. The highest relative density of 99.9% was obtained with GA powder at 48 J/mm^3^ VED and 99.7% with MAPS powder at 70 J/mm^3^.

2. Very fine microstructures consisting of lamellar α_2_/γ colonies, equiaxed γ grains, and retained β phase were obtained in all samples. Al loss during the L-PBF process led to the shift in the solidification route and resulted in the formation of the retained β phase. Increased oxygen content in the initial powder led to the formation of small oxides and an increased α_2_ volume fraction.

3. Using an increased energy input during the L-PBF with a large laser spot size resulted in an inhomogeneous microstructure consisting of Al-rich and Al-poor regions. Regions with lamellar α_2_/y colonies and equiaxed α_2_ grains surrounded with retained β phase depending on the Al concentration were found in the microstructure. There were coarse columnar and refined γ grains as a result of repetitive heating and cooling during the L-PBF.

4. The fabricated samples showed high ultimate compressive strength and strain values. The samples fabricated from GA powder demonstrated superior compressive performance compared to the samples from the MAPS powder. Both alloys showed superior compressive properties compared to the conventional TiAl-alloy.

5. The samples fabricated from the MAPS powder had higher microhardness due to the increased oxygen content and α_2_ volume fraction.

6. Further investigation will be focused on a more detailed characterization of mechanical properties at room and elevated temperatures including tensile behavior, as well as the effects of heat treatment on microstructure and properties of the TiAl-alloy.

## Figures and Tables

**Table 1:** The L-PBF process parameters used for to produce the samples.

Parameter Set	P, W	S, mm/s	HD, mm	L, mm	VED, J/mm^3^	Laser Spot Diameter, µm	Preheating Temperature, °C	Feedstock Powder
1_600	150	800	0.11	0.03	57	80	600	GA, MAPS
2_600	150	650	0.11	0.03	70			
3_600	150	1000	0.11	0.03	45			
4_600	150	1250	0.11	0.03	36			
5_600	150	950	0.11	0.03	48			
6_600	150	750	0.11	0.03	61			
1_800	150	800	0.11	0.03	57		800	
2_800	150	650	0.11	0.03	70			
3_800	150	1000	0.11	0.03	45			
4_800	150	1250	0.11	0.03	36			
5_800	150	950	0.11	0.03	48			
6_800	150	750	0.11	0.03	61			
1_900	150	800	0.11	0.03	57		900	
2_900	150	650	0.11	0.03	70			
3_900	150	1000	0.11	0.03	45			
4_900	150	1250	0.11	0.03	36			
5_900	150	950	0.11	0.03	48			
6_900	150	750	0.11	0.03	61			
Large spot	850	330	0.45	0.09	64	500	800	GA

**Table 2:** The chemical composition of mechanically alloyed plasma spheroidized (MAPS) and GA powders.

Powder	Measured by EDS				Measured by IGF
	Ti, at. %	Al, at. %	Nb, at. %	Cr, at. %	O, wt. %
MAPS	51.6 ± 0.3	43.7 ± 0.3	2.0 ± 0.1	2.2 ± 0.1	1.1 ± 0.1
GA	50.2 ± 0.2	45.7 ± 0.3	2.1 ± 0.1	2.1 ± 0.1	0.07 ± 0.01

**Table 3:** The chemical composition (in at. %) of the samples fabricated from MAPS powder at different preheating temperatures.

Preheating Temperature, °C	Ti	Al	Nb	Cr	O
600	55.2 ± 0.1	40.4 ± 0.1	2.0 ± 0.1	2.2 ± 0.1	–
800	53.8 ± 0.2	41.6 ± 0.2	2.3 ± 0.1	2.0 ± 0.2	0.14 ± 0.02
900	54.3 ± 0.1	40.9 ± 0.1	2.1 ± 0.2	2.2 ± 0.1	0.13 ± 0.02

**Table 4:** The chemical composition (in at. %) of the samples fabricated from GA powder using different laser spot diameters.

Sample	Ti	Al	Nb	Cr
Small spot, 80 μm (Sample 5_800)	50.5 ± 0.1	45.3 ± 0.2	2.1 ± 0.1	2.1 ± 0.1
Large spot, 500 μm	52.9 ± 0.2	42.3 ± 0.3	2.0 ± 0.1	2.2 ± 0.1

**Table 5:** Comparison of room temperature mechanical properties of TiAl alloy manufactured by different processes.

Material	Condition	Ultimate Compressive Strength, MPa	Compressive Strain, %
Ti-48Al-2Cr-2Nb (GA powder, this study)	As-fabricated, 900 °C preheating	2277 ± 71	32–35
Ti-48Al-2Cr-2Nb (MAPS powder, this study)	As-fabricated, 900 °C preheating	1910 ± 37 MPa	15–17
Ti-48Al-2Cr-2Nb (SEBM) [48]	As-fabricated	1800	40
Ti-48Al-2Cr-2Nb (L-PBF) [48]	As-fabricated, no preheating	612 ± 56	1,98 ± 0.55
Ti-48Al-2Cr-2Nb (SEBM) [50]	Heat-treated	2068	25
Ti-48Al-2Cr-2Nb (casted) [50]	As-fabricated	1153	~6
